# Triangulation to make decisions about what are modifiable risk factors and the new risk factors

**DOI:** 10.1002/alz.085067

**Published:** 2025-01-09

**Authors:** Naaheed Mukadam, Gill Livingston, Jonathan D Huntley, Kathy Y Liu, Sergi Costafreda Gonzalez, Geir Selbaek, Suvarna Alladi, David Ames, Sube Banerjee, Alistair Burns, Carol Brayne, Nick C Fox, Cleusa P Ferri, Laura N. Gitlin, Robert J Howard, Helen C Kales, Mika Kivimaki, Eric B Larson, Noeline Nakasujja, Kenneth Rockwood, Quincy M Samus, Kokoro Shirai, Archana Singh‐Manoux, Lon S. S. Schneider, Sebastian Walsh, Yao Yao, Andrew Sommerlad

**Affiliations:** ^1^ University College London, London United Kingdom; ^2^ University of Exeter, Exeter United Kingdom; ^3^ Division of Psychiatry, University College London, London United Kingdom; ^4^ Norwegian National Center for Ageing and Health, Vestfold Hospital Trust, Tønsberg Norway; ^5^ National Institute of Mental Health and Neurosciences [NIMHANS], Bengaluru, Karnataka India; ^6^ National Ageing Research Institute, Melbourne, VIC Australia; ^7^ University of Nottingham, Nottingham United Kingdom; ^8^ University of Manchester, Manchester United Kingdom; ^9^ Cambridge Public Health, University of Cambridge, Cambridge United Kingdom; ^10^ Universidade Federal de São Paulo (UNIFESP), São Paulo, São Paulo/SP Brazil; ^11^ Drexel University, Philadelphia, PA USA; ^12^ Division of Psychiatry, University College London, London, London United Kingdom; ^13^ University of California, Davis, Sacramento, CA USA; ^14^ University of Washington, Seattle, WA USA; ^15^ College of Health Sciences, Makerere University, Kampala Uganda; ^16^ Dalhousie University, Halifax, NS Canada; ^17^ Johns Hopkins University, Baltimore, MD USA; ^18^ Osaka University, Osaka Japan; ^19^ UCL Brain Sciences, University College London, London United Kingdom; ^20^ Keck School of Medicine of USC, Los Angeles, CA USA; ^21^ Cambridge Public Health, Cambridge United Kingdom; ^22^ Peking University, Beijing China

## Abstract

**Background:**

The 2020 Lancet Commission on dementia prevention, intervention and care estimated that up to 40% of dementia cases could be prevented by tackling 12 potentially modifiable risk factors, namely less education, hearing loss, hypertension, physical inactivity, diabetes, social isolation, excessive alcohol consumption, air pollution, smoking, obesity, traumatic brain injury, depression. As more evidence on risk factors emerges, the Lancet standing commission on dementia met to update evidence on established dementia risk factors and to consider the evidence for other risk factors.

**Method:**

We used a lifecourse approach to understand how to reduce risk or prevent dementia, as many risks operate at different timepoints in the lifespan. We considered evidence for when in the lifecourse a risk factor was relevant to development of dementia as well as the size of the effect and strength of the evidence. Our interdisciplinary, international, multicultural group of experts adopted a triangulation framework, prioritising systematic reviews and meta‐analyses, performing new meta‐analyses where needed and debated and agreed on the best available evidence and its consistency. We considered whether there was evidence of disparities in impact of risk factors based on demographic characteristics, particularly ethnicity and socioeconomic status.

**Result:**

Evidence for two new risk factors was considered strong enough to include this in our lifecourse model. We will present evidence for incorporation of these risk factors, including strength of evidence and potential mechanisms. We will also discuss risk factors for which evidence was not strong enough.

**Conclusion:**

As more evidence about risk factors emerges we can increase our understanding of how dementia develops and how to potentially prevent it. Understanding the landscape of dementia prevention research is also helpful to appreciate where further evidence is needed and what form of evidence would be most helpful in advancing our understanding.

